# Aspergillus Niger Derived Wrinkle‐Like Carbon as Superior Electrode for Advanced Vanadium Redox Flow Batteries

**DOI:** 10.1002/advs.202300640

**Published:** 2023-04-23

**Authors:** Qi Deng, Wei‐Bin Zhou, Hong‐Rui Wang, Na Fu, Xiong‐Wei Wu, Yu‐Ping Wu

**Affiliations:** ^1^ CAS Key Laboratory of Molecular Nanostructure and Nanotechnology CAS Research/Education Center for Excellence in Molecular Institute of Chemistry Chinese Academy of Sciences (CAS) Beijing 100190 P. R. China; ^2^ State Key Laboratory of Utilization of Woody Oil Resource of China Hunan Academy of Forestry Changsha Hunan 410018 P. R. China; ^3^ School of Chemistry and Materials Science Hunan Agricultural University Changsha Hunan 410128 P. R. China; ^4^ Hunan Province Yinfeng New Energy Co., Ltd. Changsha Hunan 410014 P. R. China; ^5^ College of Electrical and Information Engineering Hunan University Changsha Hunan 410082 P. R. China; ^6^ School of Energy and Environment Southeast University Nanjing 211189 P. R. China

**Keywords:** *Aspergillus Niger*, density functional theory, graphite felt, microorganism‐based electrode, vanadium redox flow batteries, wrinkle‐like carbon

## Abstract

The scarcity of high electrocatalysis composite electrode materials has long been suppressing the redox reaction of V(II)/V(III) and V(IV)/V(V) couples in high performance vanadium redox flow batteries (VRFBs). Herein, through ingeniously regulating the growth of *Aspergillus Niger*, a wrinkle‐like carbon (WLC) material that possesses edge‐rich carbon, abundant heteroatoms, and nature wrinkle‐like structure is obtained, which is subsequently successfully introduced and uniform dispersed on the surface of carbon fiber of graphite felt (GF). This composite electrode presents a lower overpotential and higher charge transfer ability, as the codoped multiheteroatoms increase the electrocatalysis activity and the wrinkled structure affords more abundant reaction area for vanadium ions in the electrolyte when compared with the pristine GF electrode, which is also supported by the density functional theory (DFT) calculations. Hence, the assembled battery using WLC electrodes achieves a high energy efficiency of 74.5% for 300 cycles at a high current density of 200 mA cm^−2^, as well as the highest current density of 450 mA cm^−2^. The WLC material not only uncovers huge potential in promoting the application of VRFBs, but also offers referential solution to synthesis microorganism‐based high‐performance electrode in other energy storage systems.

## Graphical abstract

By controlling the growth of *Aspergillus Niger* and the distribution of its products on graphite felt (GF), this work exhibits a microorganism‐based high‐performance electrode, which possesses edge‐rich carbon, abundant heteroatoms, and wrinkle‐like structure and demonstrates excellent electrocatalytic activity for V(II)/V(III) and V(IV)/V(V) redox couples in vanadium redox flow batteries(VRFBs), which is supported by density functional theory (DFT) calculations and experiment analyses.

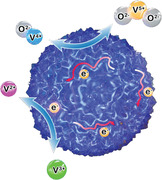

## 1 Introduction

Among various remarkable energy storage technologies as solid supplements to smart grids, vanadium redox flow batteries (VRFBs) stand out with low price, high safety, high output, and long life.^[^ ^1^ ^]^ However, the applications of VRFBs are seriously hindered by the low electrocatalysis ability to the reaction of V(II)/V(III) and V(IV)/V(V) redox couples. Hence, it's urgent to develop overall high‐performance electrode materials with satisfying electrocatalysis activity.

The carbon materials have been performing an important role in energy storage and conversion systems, as they are low‐cost, wildly sourced, environmentally friendly, and easily regulated in structure and morphology.^[^ ^2^ ^]^ Remarkably, graphite felt (GF) is the representative one due to its excellent advantages such as leading conductivity, three‐dimensional interconnecting conducting networking, abundant active sites for redox reactions, and large surface area, and has been widely used in VRFBs.^[^ ^3^ ^]^ Even so, researchers still have paid attention to boosting the performance of GF to endow it with higher electrocatalytic activity to the reaction of V(II)/V(III) and V(IV)/V(V) redox couples, such as doping with heteroatoms as N, P, and S,^[^ ^4^ ^]^ activating with acids as H_2_SO_4_ and H_3_PO_4_,^[^ ^5^ ^]^ and fabricating multidimensional composite materials with ingenious morphologies.^[^ ^6^ ^]^ More specifically, doping has been acknowledged by the academic society as an effective method, as doping can improve the conductivity for ions and electrons, introduce a large number of active defect sites, enlarge the specific surface area, and import sufficient electroactive functional groups, which all contribute to the improvement on the reaction of V(II)/V(III) and V(IV)/V(V) redox couples. And many valuable works have been proposed to constructing high‐performance electrodes doped with heteroatoms homogeneously. For example, Wei et al. reported that benefiting from the uniform distribution of nitrogen elements on the nanoarrays, the well‐distributed nucleation of Li ions and loading of S was greatly enhanced;^[^ ^7^ ^]^ Qu et al. demonstrated that owing to the homogeneous introduction of nitrogen, the sodium metal anode was stabilized with decreased nucleation energy barrier and stabilized SEI;^[^ ^8^ ^]^ Xin et al. revealed that the uniformly distributed nitrogen functional groups from doping in the carbon nanofibers afford homogeneous sites for the nucleation of lithium ions, leading to uniform deposition of lithium.^[^ ^9^ ^]^ No doubt, these materials have demonstrated extraordinary performance by giving full play to the advantages of the sophisticated structures, especially of the advantages of homogeneous doping of heteroatoms. Besides, there also are many vitally important works that uncovered the significance of uniform distribution of imported heteroatoms or nanoparticles onto the framework of the substrate.^[^ ^10^ ^]^ For instance, Zhao et al. revealed that the uniform distributed nanoparticles could induce much improved electroactive surface area;^[^ ^11^ ^]^ Kim et al. concluded that through uniform electrodeposition, Bi^3+^ was homogeneously distributed onto the porous electrode, which played an vital role in enhancing the performance of iron‐chromium redox flow batteries;^[^ ^12^ ^]^ Liu et al. demonstrated that the uniform distributed sulfur functional group could induce uneven electrons, which boosted redox reactions.^[^ ^4a^ ^]^ In short, these works expounded that uniform distribution of imported heteroatoms was responsible for the enhanced electroactivity of the prepared electrodes, and finally exhibited excellent overall properties in batteries.

Inspired by the design of homogeneous dope, herein we propose a novel wrinkle‐like carbon (WLC), which was derived from *Aspergillus Niger* and able to advance the redox reactions of vanadium ions in VRFBs. During the growth process of *Aspergillus Niger*, huge number of polysaccharide wrapped spores with high‐speed ejection and large critical motion distance were produced, namely the precursor of WLC,^[^ ^13^ ^]^ which are uniform distributed and attached onto the surface of carbon fibers of the GF. The spores possess a large effective specific surface area and a microsized cell structure that is made from the wrinkled cell wall. This cell structure is mainly comprised of organic frameworks, containing carbon, oxygen, nitrogen, phosphorus, and sulfur elements. During the anneal process, the carbon from the organic framework was chemically transformed into WLC material. As the WLC material possesses uniform distribution of heteroatoms, wrinkle‐like structure, and edge‐rich carbon, it presents fast electron transfer between the interface and surface, exhibits high electrocatalysis toward vanadium ions redox couples, and apparently reduces the overpotential during the oxidation and reduction process. As a result, the WLC material exhibited a high energy efficiency of 74.5% for 300 cycles at a high current density of 200 mA cm^−2^, as well as the highest current density of 450 mA cm^−2^. The abovementioned excellent performance of WLC has not only supported it as a promising electrode to promote the large‐scale development of VRFBs, but also highlight the prior advantages of using fungus to fabricate in situ doped composite electrodes for other energy storage systems.

## 2 Results and Discussion

**Figure 1:**
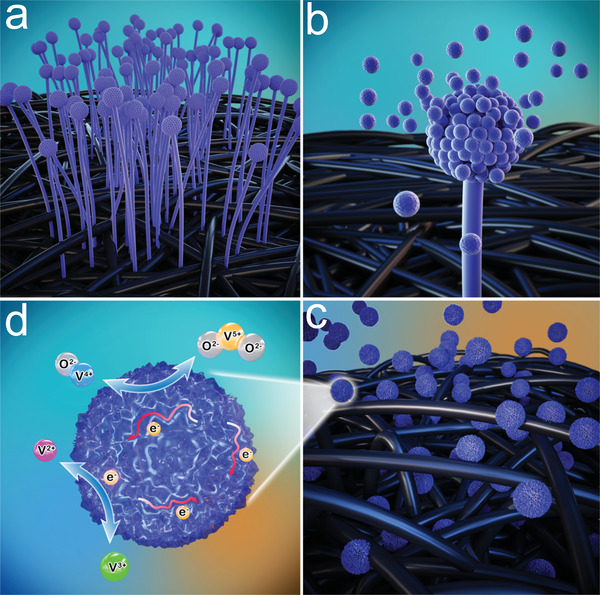
Schematic illustration of the WLC. a) *Aspergillus Niger* growing on the carbon fiber. b) Spores launching. c) The WLC distribution on the carbon fiber. d) The reaction mechanism of WLC electrodes in VRFBs with enlarged view.

To obtain the WLC electrodes, the neonatal *Aspergillus Niger* was placed in an incubator at 30 °C and cultured for 72 h. The evolve process of the WLC electrode is shown in **Figure** **1**, and the culturing images of the WLC electrode precursor at different stages are depicted in Figure S1 (Supporting Information). Specifically, after culturing for 24 h (Figure S1a, Supporting Information), coffee substances coating on black GF can be obviously observed, which implied that *Aspergillus Niger* in situ grew onto the surface of carbon fiber of GF (Figure 1a). After culturing for 36 h with the image shown in Figure S1b (Supporting Information), part of coffee substances turned white with cotton‐shaped morphology, which indicated the intermediate propagation of *Aspergillus Niger*. When cultured for another 12 h with the image shown in Figure S1c (Supporting Information), more coffee substances and many dispersive particles can be observed with less white substances, which indicated the continuous propagation of *Aspergillus Niger* with a large quantity of spores launched (Figure S2, Supporting Information). Finally, at the end of cultivation process for 72 h with the image shown in Figure S1d (Supporting Information), coffee substances and particles turned into mocha while the color of white cotton‐shaped substances changed to lilac, which demonstrated that the spores were produced on the top of mature cell and launched around with the assistance of the turgor pressure within the cell (Figure 1b).^[^ ^14^ ^]^ The launched spores were scattered within the space of GF, and adhered toughly to the surface of carbon fiber through polysaccharide of the cell wall (Figure S1d, Supporting Information). Finally, the WLC electrodes were obtained from the WLC, which was produced by annealing spores that were uniform distributed on the surface of carbon fiber of GF (Figure 1c). Besides, the reaction mechanism of WLC electrodes in VRFBs with enlarged view was depicted in Figure 1d. As a contrast, the pristine GF without further purification was used as electrode, denoted as PLC.

**Figure 2:**
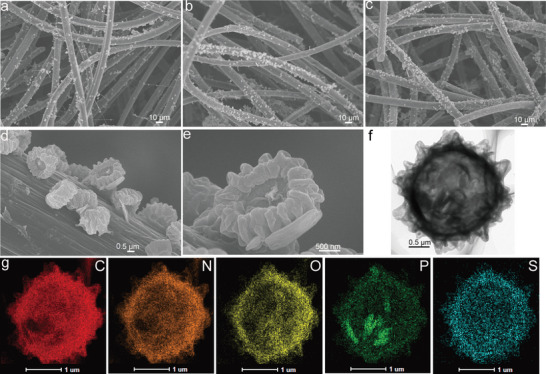
Scanning electron microscope transmission electron microscope (SEM–TEM) images of WLC from *Aspergillus Niger*. a–e) SEM images and f) TEM image of WLC. g) Energy dispersive X‐ray spectroscopy (EDX) elemental mapping images of WLC with carbon (C), nitrogen (N), oxygen (O), phosphorus (P), and sulfur (S).

To investigate the distribution of WLC on the surface of GF, detection on three different parts from the same GF was inspected using scanning electron microscope (SEM), as shown in **Figure** **2**a–c.

The WLC materials presented a high consistency of uniform distribution in the axial and radial direction on carbon fiber of GF. In addition, it is obvious that WLC and carbon fiber is chemical coupled by the carbon derived from viscous polysaccharide on the cell wall (Figure 2d,e). The WLC material possesses large effective reaction area and electrolyte contact area because it is made up of folded and continuous carbon (Figure 2f and Figure S3, Supporting Information). What's more, the elemental distribution of WLC material was detected by energy dispersive X‐ray spectroscopy (EDX) (Figure 2g). As shown in Figure 2g, the WLC is constituted of five elements, namely carbon, nitrogen, oxygen, phosphorus, and sulfur, which is attributed to the micro/nanocell structure of the WLC and uniformly distributed in the framework of the WLC, affording extra active defect sits on the surface of GF for the attraction and adsorption of vanadium ions.^[^ ^15^ ^]^

**Figure 3:**
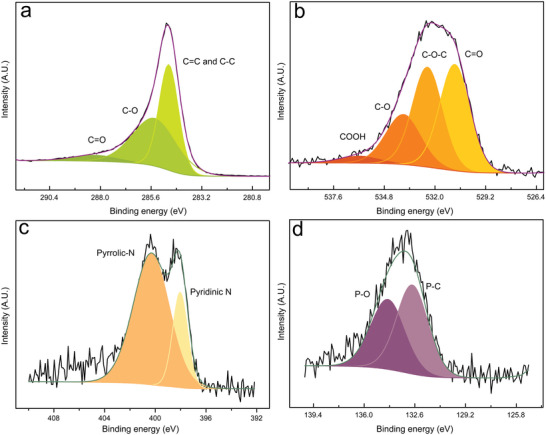
X‐ray photoelectron spectroscopy (XPS) spectra for a) C 1s, b) O 1s, c) N 1s, and d) P 2p peaks and the high‐resolution fitting of WLC.

To dig deep into the information of structure and components, the WLC material was detected by X‐ray photoelectron spectroscopy (XPS), and the chemical bond and valence states are derived from high‐resolution spectra. As shown in **Figure** **3** and Figure S4 (Supporting Information), the WLC material possesses abundant heteroatoms, such as oxygen (O), nitrogen (N), and phosphorus (P), owing to the special cell component of fungal propagules.

Figure 3a shows three peaks of C 1s at 284.76, 285.42, and 288.23 eV, corresponding to the chemical bond of C=C/C−C, C−O, and C=O, respectively.^[^ ^16^ ^]^ The spectra of O 1s display four major peaks at 530.91 eV (C=O), 532.42 eV (C−O−C), 533.74 eV (C−O), and 536.14 eV (COOH) (Figure 3b).^[^ ^17^ ^]^ The high‐resolution XPS spectra of N 1s present two major peaks at 398.25 and 400.96 eV, attributed to the pyridinic‐N and pyrrolic‐N, respectively (Figure 3c).^[^ ^18^ ^]^ On the basis of abundant reported literatures,^[^ ^2^, ^19^ ^]^ the nitrogen functional groups are considered as a catalyst and accelerating the reduction and oxidation reactions on account of the benefits from the donation of nitrogen atoms and positively charging the carbon atoms around the doped nitrogen atoms.^[^ ^20^ ^]^The P 2p region is divided into two species with a binding energy of 132.77 and 134.41 eV, corresponding to P−C and P−O, respectively (Figure 3d).^[^ ^21^ ^]^ Generally, the doping of phosphorus is in favor of the content promotion of oxygen‐containing function groups though the P−O bonds,^[^ ^22^ ^]^ which increase the number of catalytic binding sites of the WLC for the reaction of vanadium redox couples and eventually improves the performance of vanadium redox flow batteries. The two peaks of sulfur at 164.42 and 168.24 eV are not obvious, corresponding to C−S and O−S chemical bond, respectively (Figure S4b, Supporting Information).^[^ ^23^ ^]^ In contrast, the PLC electrode only presents peaks of C, O, and N in the XPS spectra (Figures S4b and S5, Supporting Information).

**Figure 4:**
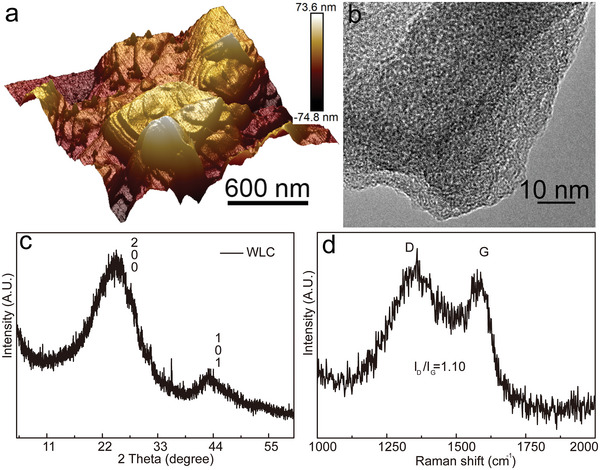
The typical spectra of a) 3D atomic force microscopy (AFM), b) transmission electron microscope (TEM), c) X‐ray diffraction (XRD), and d) Raman of WLC.

Furthermore, the spectrogram of atomic force microscopy (AFM) (**Figure** **4**a) presents an edge‐plane and basal‐plane carbon of WLC material, which are related with the effective active sites and electrochemistry catalysis activity.

Comparing to the basal‐plane carbon, the edge‐plane carbon possesses faster charge transfer and higher electrocatalysis activity.^[^ ^24^ ^]^ The edge‐plane was observed on the surface of WLC, which is like steep cliff of a mountain located at different altitude, so that the exposed carbon provides enough reactive sites for vanadium ions. The transmission electron microscope (TEM) image also shows obvious gradient change of the WLC carbon layer, as shown in Figure 4b. To deeply investigate the phase properties of WLC, the X‐ray diffraction (XRD) and Raman analysis were carried out and shown in Figure 4c,d, respectively. The pattern of XRD exhibits two peaks located at 24.8° and 43.1°, which are corresponding to the (002) and (101) planes, indicating an amorphous carbon and hexagonal graphitic carbon, respectively.^[^ ^25^ ^]^ The Raman spectrum demonstrates two characteristic peaks of 1359 and 1575 cm^−1^, corresponding to the defects/disorder carbon (D band) and graphitic carbon (G band), respectively. The intensity ratio (*I*_D_/*I*_G_) of D band and G band can estimate the disorder and order for carbon degree in the WLC.^[^ ^26^ ^]^

**Figure 5:**
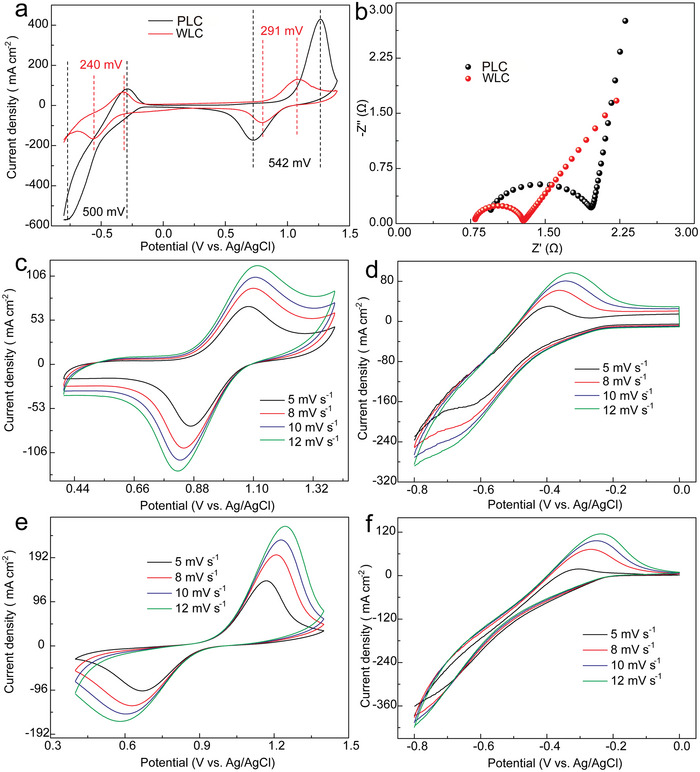
a) Cyclic voltammetry (CV) test, b) electrochemical impedance spectroscopy (EIS) spectra of the PLC and WLC electrodes at 10 mV s^−1^. c–f) CV test for WLC and PLC electrodes at 5, 8, 10, and 12 mV s^−1^.

The peak potential difference (△*E*) and the ratio (*I*_pa_/*I*_pc_) of peak current density were important indexes to evaluate the electrocatalysis activity of electrode toward V(II)/V(III) and V(IV)/V(V) redox couples in cyclic voltammetry (CV) test. **Figure** **5**a presents the difference in electrocatalytic activity of positive and negative electrolytes from PLC and WLC electrodes. The peak potential difference between the cathode and anode of WLC is 283 and 250 mV for the positive and negative half‐cell, respectively, which are lower than that of the PLC (540 and 497 mV, respectively).

What's more, the peak potential difference of the WLC positive and negative electrode are decreased by 90.8% and 98.8%, respectively, compared with the PLC electrodes (Table S1, Supporting Information), indicating that the overpotential was largely decreased as the current density was dispersed by edge‐rich carbon and WLC.^[^ ^27^ ^]^ This is ascribed to the uniform distribution of heteroatoms dopes, which could provide ample active defect sites, lager specific surface area, and improved conductivity.^[^ ^28^ ^]^ Moreover, the ratio of current density from the WLC electrode is close to 1 in positive and negative electrolyte during the reaction process, indicating higher electrochemical activity and redox reversibility than the PLC electrode. The electron transfer rate and electrochemistry activity were investigated using electrochemical impedance spectroscopy (EIS) test, which was used to test the Randles circuit of the PLC and WLC electrodes. As shown in Figure 5b, the radius of the semicircle is an evaluation on charge transfer between the surface of electrode and vanadium ions in the high frequency region,^[^ ^29^ ^]^ which is assigned to R2 in the equivalent circuit (Figure S6, Supporting Information). The values of R2 from the PLC and WLC electrodes are 1.06 ± 0.01 and 0.48 ± 0.02 Ω, indicating that the WLC material accelerates the electron transfer from the electrolyte to the surface of WLC composite electrode, which is ascribed to the uniform distribution of abundant heteroatoms on the material surface as the electrical conductivity of the WLC for electrons and ions could be boosted, owing to the donated electrons from the heteroatoms into carbon system of GF fibers and reduced band gap of doped carbon.^[^ ^20^ ^]^ The evaluation on the electrocatalytic activity of WLC material was also carried out by conducting CV at different scan rates. The WLC and PLC electrodes present increased peak current densities with the augment of scan rates. Besides, the CV curves of WLC electrode still maintain four clearly peaks of reduction and oxidation reactions in positive and negative electrolyte at different scan rates. The peak potential difference and the ratio of current density of WLC electrode retain at a high level of stability while the scan rates are between 5 and 12 mV s^−1^ (Figure S7, Supporting Information). The abovementioned electrochemistry activities are mainly credited to the decoration of the WLC material on the surface of carbon fiber, which provides a wrinkle‐structured carbon with rich defects and edge carbon to accelerate the reactions process of vanadium ions during the liquid and solid surface.^[^ ^30^ ^]^

**Figure 6:**
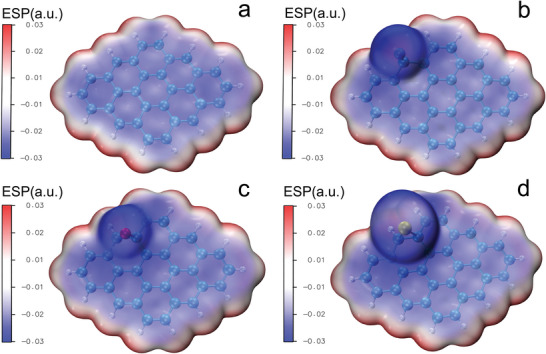
ESPdistribution on the Van der Waals surface of a) PLC without heteroatoms and b) WLC with nitrogen atoms; c) WLC with oxygen atoms; d) WLC with sulfur atoms.

Furthermore, in order to explore the inherent advantages of WLC, density functional theory (DFT) calculations were employed. With the assistance of the multifunctional wavefunction analyzer, Multiwfn,^[^ ^31^ ^]^ the electrostatic potential (ESP)^[^ ^32^ ^]^ distribution of PLC and WLC was displayed in **Figure** **6** and Figure S8 (Supporting Information). As shown in Figure 6a and Figure S8 (Supporting Information), except for the edge pink region representing positive ESP value of PLC, the central blue region revealed negative ESP values, which are possible adsorption sites and attractive for the V(II)/V(III) and V(IV)/V(V) redox couples.^[^ ^33^ ^]^ What's more, as shown in Figure 6b–d, owing to the heteroatoms from the spore precursors, such as nitrogen, oxygen, and sulfur, circular dark blue region with high ESP value can be observed, which illustrated possible stronger attraction for the V(II)/V(III) and V(IV)/V(V) redox couples of WLC than the PLC.^[^ ^3^, ^34^ ^]^ Besides, as shown in Figure S8 (Supporting Information), the smaller relative value of the ESP with baby blue distribution from the phosphorus‐doped WLC than that from the systems with nitrogen, oxygen, and sulfur can be observed, which indicates that the phosphorus in this system could donate less electronics to the system.^[^ ^34^ ^]^ During the charging and discharging process for the redox couples, less energy was consumed, which was confirmed by the binding energy, as shown in Tables S2–S5 (Supporting Information).

**Figure 7:**
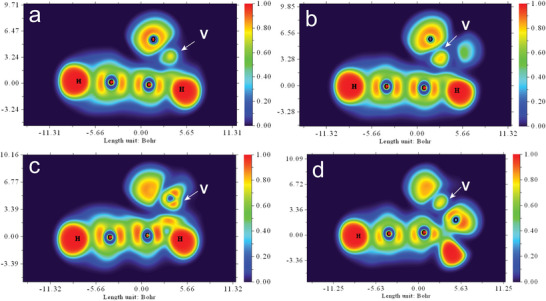
Electron localization function (ELF) plane of a) PLC and c) WLC with VO^2+^; b) PLC and d) WLC with VO_2_^+^.

Specifically, the binding energy value for WLC with V^2+^ was 0.4221 a.u., which is higher than 0.3621 a.u. of PLC. Besides, the systems binding V^3+^, VO^2+^, and VO_2_^+^ showed the same trends as shown in Tables S3–S5 (Supporting Information), which reflected the acceleration of heteroatoms for the V(II)/V(III) and V(IV)/V(V) redox couples and finally boosted the electrochemical performance of VRFBs.^[^ ^28h^ ^]^ What's more, in order to explore the adsorption mechanism of vanadium on different substrates materials, the electron localization function (ELF)^[^ ^35^ ^]^ plane of PLC and WLC with VO^2+^ and VO_2_^+.^was shown in **Figure** **7**. As the electron distribution of V atom (VO^2+^) of PLC shown in Figure 7a, green region with electron localization value of approximately 0.55 and buff region with electron localization value of approximately 0.72 in the center was observed. When in Figure 7c for WLC, dark blue region with electron localization value of approximately 0.15 was observed in the center of the vanadium atom. Around the center, there were deep yellow regions with electron localization value of approximately 0.85. And the region between the oxygen and vanadium atom was azure, with electron localization value of approximately 0.40, indicating lower electron localization between the oxygen and vanadium atom. As for VO_2_^+^ shown in Figure 6b,d, the same trend can be observed. In Figure 7b for PLC with VO_2_^+^, green region with electron localization value of approximately 0.5 distributed in the edge and deep yellow regions with electron localization value of approximately 0.78 were located in the center. However, in Figure 7d for PLC with VO_2_^+^, the electron localization value of the deep yellow regions in the center was approximately 0.70, lower than that of PLC. As shown in Figure S9 (Supporting Information), the same trends of V^2+^ and V^3+^ can be observed. These results implied that with the assistance of oxygen atom from the precursor, the electron exchange capability of vanadium atom was enhanced, finally boosting the reaction activities of the V(II)/V(III) and V(IV)/V(V) redox couples.

**Figure 8:**
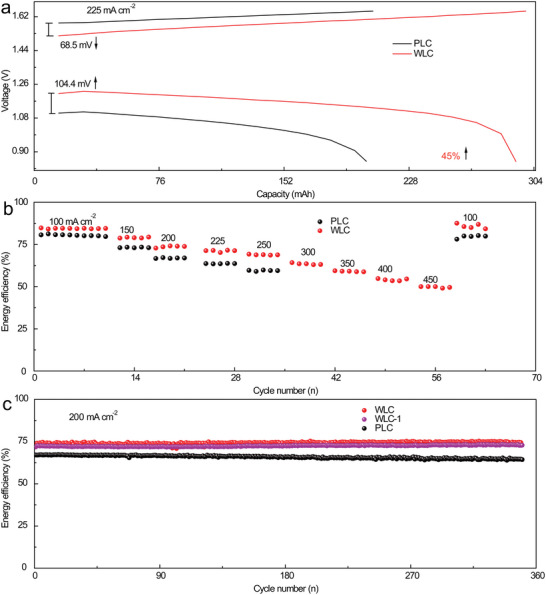
The PLC and WLC electrodes were used in vanadium redox flow batteries (VRFBs). a) voltage profile at 225 mA cm^−2^, b) energy efficiency at different current densities, c) energy efficiency of long cycling at 200 mA cm^−2^.

For deeper investigation on electrochemical properties of the WLC material, VRFBs were assembled and tested in various conditions. The voltage profiles of the WLC and PLC electrodes were obtained at 225 mA cm^−2^, as shown in **Figure** **8**a. While the assembled batteries operated at high current density, the battery with WLC electrode exhibited a lower charge voltage and a higher discharge voltage compared with the one with PLC electrode, which is the key to enhance the energy output quality of VRFBs.

Owing to the depression on overpotential, the battery assembled with WLC electrode showed an improvement of discharge capacity by 45% compared with the one using PLC electrode (Figure 8a). The maximal current density of 250 and 450 mA cm^−2^ was achieved from the battery using the PC and WLC electrodes, respectively, which also demonstrated steady energy efficiency of the WLC electrodes at different current density. The difference of energy efficiency between the PLC and WLC electrodes was increased with the increase of current densities, which is attributed to the fast electron transfer at vanadium ion‐electrode interfaces and efficient catalytic activity of WLC material from the heteroatoms dopes.^[^ ^19f^ ^]^ In order to evaluate the stability of the WLC and PLC electrodes during long‐time cycles, the assembled cells were operated at high current density of 200 mA cm^−2^ for 350 cycles. Two types of electrodes derived from the parallel experiment were named as WLC and WLC‐1, which presented ultrastable and much the same energy efficiency without decline during long cycles. On the contrary, the battery using the PLC electrode showed lower energy efficiency that then decreased with the increased cycles compared with the battery with WLC electrodes, which is ascribed to low electron transfer and electrocatalytic activities of the PLC electrode toward vanadium ions.

## 3 Conclusion

In summary, we designed a WLC materials used as composite electrode to accelerate the redox reaction of vanadium ions in VRFBs through controlling the growth of *Aspergillus Niger*, and succeed in the uniform distribution of biocarbon onto the carbon fiber of GF. Moreover, on account of the homogeneous distributed multi‐heteroatoms and edge‐carbon of WLC materials, vanadium ions presented a higher electrochemical activity and lower overpotential for the reactions in anode and cathode, which were also revealed by the DFT and in accordance with the analyses. The micro/nanostructure of WLC served as the bridge connection between vanadium ions and electrode, and it improved the transfer of electrons between the interface and surface. As a result, the WLC electrodes delivered a high energy efficiency of 74.5% for 300 cycles at an ultrahigh current density of 200 mA cm^−2^, as well as the highest current density of 450 mA cm^−2^. The WLC materials with splendid performance not only have been proved to be a promising electrode material to promote the application of high‐performance VRFBs, but also can be applied in other energy storage system with excellent electrochemical performance.

## 4 Experimental Section

### Preparation of the Liquid Culture Media

An amount of 50 g wheat bran and 500 mL water were put into a enamel cup to form a mixture, then it was heated in an electromagnetic furnace with continuous stirring until boiling for 15 min. Finally, it was filtered to remove the solid.^[^ ^36^ ^]^

### Fabrication of the WLC Electrode

The GF (3 × 4 cm) was immersed in the abovementioned liquid culture media and dispersed with ultrasound for 15 min, and dried at 90 °C in a baking oven for 12 h. The prepared GF was inoculated with the seed liquid of *Aspergillus Niger* at OD_600_ = 0.5 (7.00 mL) and grown at 30 °C for 72 h in an incubator, then the WLC electrode precursor was fabricated (Figure S1, Supporting Information). After that, the precursor materials were dried in a drying oven for 12 h and annealed at 800 °C for 1 h under argon atmosphere. The prepared electrodes were washed by deionized water for five times and dried in a baking oven until the weight of electrode remains constant.

Characterization of the materials and electrodes

Their micro/nanostructure, elements distribution, and composition were detected by a SEM (S4800, Hitachi, Japan), TEM (2100F, JEOL, Japan), XRD (X' Pert PRO MPD, PANalytical, Netherlands), Raman spectra (inVia, Renishaw, England), an atomic force microscope (AFM, NanomanVs, Veeco, USA), and XPS (K‐Alpha+, Thermo Fisher Scientific, USA).

### Electrochemical Measurements

All the electrochemical characterizations were conducted at room temperature under the atmosphere of air. Nafion 115 (Dupont, USA) was employed as membrane and the flow rate of the electrolyte was set to 15 mL min^−1^ without flow field. The electrochemical performances of the PLC and WLC electrodes were investigated using an electrochemical working station (CHI 604E, ChenHua, China) equipped with CV and EIS, and the results of EIS were simulated by Zview software. In this part, the WLC and PLC electrode with the size of 5 × 5 mm, the platinum sheet and Ag/AgCl electrode was used as the working, counter, and reference electrode, respectively. Besides, the solution comprising 3 m H_2_SO_4_ and 0.05 m VOSO_4_ was used as the electrolyte. When conducting the electrochemical characterization of long‐cycle charge–discharge, the GF with a size of 3 × 4 cm without further purification and the WLC electrode after the fabrication with the same size was used as the PLC and WLC working electrode, respectively. The batteries were tested using 15 mL electrolyte comprising 0.375 m V_2_(SO_4_)_3_, 0.75 m VOSO_4_, and 3.5 m H_2_SO_4_ and cell testing system (CT2001A, Wuhan Land Electronics Co., Ltd., China).

### DFT Calculation

On the basis of DFT, all the caculation data was output by using the computional chemistry package of Gaussian 16^[^ ^37^ ^]^ with the method of B3LYP^[^ ^38^ ^]^ and the basis set of 6–311G(d,p).^[^ ^39^ ^]^ The analysis on ESP and ELF was conducted by using the multifunctional wavefuntion analyzer, Multiwfn. The binding enery (*E*_b_) for different systems of PLC and WLC was calculated following the equation below:

(1) $$E_{b}=E_{1}+E_{2}-E_{\mathrm{total}}$$

where *E*_total_, *E*_1_, and *E*_2_ represent the energy of the absorbed systems, different vanadium ions with different valence state, and the different substrate materials (PLC and WLC), respectively.

## Conflict of Interest

The authors declare no conflict of interest.

## Supporting information

Supporting Information

## Data Availability

Research data are not shared.

## References

[1] a) B. M. Sivakumar , V. Prabhakaran , K. Duanmu , E. Thomsen , B. Berland , N. Gomez , D. Reed , V. Murugesan , ACS Appl. Energy Mater. 2021, 4, 6074;

[2] a) W. Li , Z. Zhang , Y. Tang , H. Bian , T.‐W. Ng , W. Zhang , C.‐S. Lee , Adv. Sci. 2016, 3, 1500276; 10.1002/advs.201500276 PMC5064734 27774399

[3] a) R. Fang , K. Chen , L. Yin , Z. Sun , F. Li , H. M. Cheng , Adv. Mater. 2019, 31, 1800863; 10.1002/adma.201800863 29984484

[4] a) Z. Xu , M. Zhu , K. Zhang , X. Zhang , L. Xu , J. Liu , T. Liu , C. Yan , Energy Storage Mater. 2021, 39, 166;

[5] Z. He , Y. Lv , T. Zhang , Y. Zhu , L. Dai , S. Yao , W. Zhu , L. Wang , Chem. Eng. J. 2022, 427, 131680.

[6] a) Q. Deng , X.‐Y. HuangYang , X. Zhang , Z.‐H. Xiao , W.‐B. Zhou , H.‐R. Wang , H.‐Y. Liu , F. Zhang , C.‐Z. Li , X.‐W. Wu , Y.‐G. Guo , Adv. Energy Mater. 2022, 12, 2103186;

[7] R. Lu , M. Cheng , L. Mao , M. Zhang , H. Yuan , K. Amin , C. Yang , Y. Cheng , Y. Meng , Z. Wei , EcoMat 2020, 2, e12010.

[8] B. Liu , D. Lei , J. Wang , Q. Zhang , Y. Zhang , W. He , H. Zheng , B. Sa , Q. Xie , D.‐L. Peng , B. Qu , Nano Res. 2020, 13, 2136.

[9] M. Shu , X. Li , L. Duan , M. Zhu , X. Xin , Nanoscale 2020, 12, 8819. 32250382 10.1039/d0nr01111h

[10] a) X. Zhang , D. Zhang , Z. Xu , K. Zhang , Y. Zhang , M. Jing , L. Liu , Z. Zhang , N. Pu , J. Liu , C. Yan , Chem. Eng. J. 2022, 439, 135718;

[11] H. R. Jiang , W. Shyy , M. C. Wu , L. Wei , T. S. Zhao , J. Power Sources 2017, 365, 34.

[12] Y. Ahn , J. Moon , S. E. Park , J. Shin , J. Wook Choi , K. J. Kim , Chem. Eng. J. 2021, 421, 127855.

[13] R. Marcus , S. Agnese , M. M. Bandi , C. Ann , H. R. Dillard , P. Anne , Proc. Natl. Acad. Sci. U. S. A. 2010, 107, 17474. 20880834 10.1073/pnas.1003577107 PMC2955148

[14] M. Roper , R. E. Pepper , M. P. Brenner , A. Pringle , Proc. Natl. Acad. Sci. U. S. A. 2008, 105, 20583. 19104035 10.1073/pnas.0805017105 PMC2634873

[15] Q. Li , A. Bai , Z. Xue , Y. Zheng , H. Sun , Electrochim. Acta 2020, 362, 137223.

[16] a) W. Chen , D. Li , L. Tian , W. Xiang , T. Wang , W. Hu , Y. Hu , S. Chen , J. Chen , Z. Dai , Green Chem. 2018, 20, 4438;

[17] a) J. Cherusseri , K. K. Kar , J. Mater. Chem. A 2016, 4, 9910;

[18] a) Q. Wang , L. Shang , R. Shi , X. Zhang , Y. Zhao , G. I. N. Waterhouse , L.‐Z. Wu , C.‐H. Tung , T. Zhang , Adv. Energy Mater. 2017, 7, 1700467;

[19] a) Z. He , M. Li , Y. Li , L. Wang , J. Zhu , W. Meng , C. Li , H. Zhou , L. Dai , Appl. Surf. Sci. 2019, 469, 423;

[20] D.‐S. Yang , J. H. Han , J. W. Jeon , J. Y. Lee , D.‐G. Kim , D. H. Seo , B. G. Kim , T.‐H. Kim , Y. T. Hong , Mater. Today Energy 2019, 11, 159.

[21] a) J. Zhang , L. Dai , Angew. Chem., Int. Ed. 2016, 128, 13490;

[22] W. Ling , Z.‐A. Wang , Q. Ma , Q. Deng , J.‐F. Tang , L. Deng , L.‐H. Zhu , X.‐W. Wu , J.‐P. Yue , Y.‐G. Guo , Chem. Commun. 2019, 55, 11515. 10.1039/c9cc05355g 31495839

[23] S. Yuan , Z. Guo , L. Wang , S. Hu , Y. Wang , Y. Xia , Adv. Sci. 2015, 2, 1500071. 10.1002/advs.201500071 PMC5115426 27980964

[24] W. Yuan , Y. Zhou , Y. Li , C. Li , H. Peng , J. Zhang , Z. Liu , L. Dai , G. Shi , Sci. Rep. 2013, 3, 2248. 23896697 10.1038/srep02248 PMC3727060

[25] D. Yuan , J. Chen , J. Zeng , S. Tan , Electrochem. Commun. 2008, 10, 1067.

[26] H. Hou , X. Qiu , W. Wei , Y. Zhang , X. Ji , Adv. Energy Mater. 2017, 7, 1602898.

[27] L. Tao , M. Qiao , R. Jin , Y. Li , Z. Xiao , Y. Wang , N. Zhang , C. Xie , Q. He , D. Jiang , G. Yu , Y. Li , S. Wang , Angew. Chem., Int. Ed. Engl. 2019, 58, 1019. 30479055 10.1002/anie.201810207

[28] a) Z. He , L. Shi , J. Shen , Z. He , S. Liu , Int. J. Energy Res. 2015, 39, 709;

[29] S. Yang , X. Feng , L. Zhi , Q. Cao , J. Maier , K. Mullen , Adv. Mater. 2010, 22, 838. 20217794 10.1002/adma.200902795

[30] Z. Liu , Z. Zhao , Y. Wang , S. Dou , D. Yan , D. Liu , Z. Xia , S. Wang , Adv. Mater. 2017, 29, 1606207. 10.1002/adma.201606207 28276154

[31] T. Lu , F. Chen , J. Comput. Chem. 2012, 33, 580. 22162017 10.1002/jcc.22885

[32] T. Lu , S. Manzetti , Struct. Chem. 2014, 25, 1521.

[33] a) X. Ni , C. Wang , Y. Su , Y. Luo , Y. Ye , X. Su , W. He , S. Wang , Y. Hong , Y. Chen , G. Zhou , B. Liu , Nanotechnol. Rev. 2022, 11, 1209;

[34] Q. Ma , X.‐X. Zeng , C. Zhou , Q. Deng , P.‐F. Wang , T.‐T. Zuo , X.‐D. Zhang , Y.‐X. Yin , X. Wu , L.‐Y. Chai , Y.‐G. Guo , ACS Appl. Mater. Interfaces 2018, 10, 22381. 29902919 10.1021/acsami.8b04846

[35] T. Lu , F.‐W. Chen , Acta Phys. ‐ Chim. Sin. 2011, 27, 2786.

[36] S. Martins , S. I. Mussatto , G. Martinez‐Avila , J. Montanez‐Saenz , C. N. Aguilar , J. A. Teixeira , Biotechnol. Adv. 2011, 29, 365. 21291993 10.1016/j.biotechadv.2011.01.008

[37] M. J. Frisch , G. W. Trucks , H. B. Schlegel , G. E. Scuseria , M. A. Robb , J. R. Cheeseman , G. Scalmani , V. Barone , G. A. Petersson , H. Nakatsuji , X. Li , M. Caricato , A. V. Marenich , J. Bloino , B. G. Janesko , R. Gomperts , B. Mennucci , H. P. Hratchian , J. V. Ortiz , A. F. Izmaylov , J. L. Sonnenberg , D. Williams‐Young , F. Ding , F. Lipparini , F. Egidi , J. Goings , B. Peng , A. Petrone , T. Henderson , D. Ranasinghe , et al., *Gaussian 16 Rev. C.02*, Gaussian Inc., Wallingford, CT 2016.

[38] P. J. Stephens , F. J. Devlin , C. F. Chabalowski , M. J. Frisch , J. Phys. Chem. 1994, 98, 11623.

[39] a) G. W. Spitznagel , T. Clark , P. von Ragué Schleyer , W. J. Hehre , J. Comput. Chem. 1987, 8, 1109;

