# Supplementary material for: Aspergillus Niger Derived Wrinkle‐Like Carbon as Superior Electrode for Advanced Vanadium Redox Flow Batteries
Source: Adv Sci (Weinh). 2023 Apr 23;10(18):2300640. doi: 10.1002/advs.202300640 (PMC10288236; doi:10.1002/advs.202300640)
Supplement: Supplementary file 1 — Supporting Information [file ADVS-10-2300640-s001.pdf]

ADVANCED SCIENCE
Open Access

Supporting Information

for *Adv. Sci.*, DOI 10.1002/advs.202300640

Aspergillus Niger Derived Wrinkle-Like Carbon as Superior Electrode for Advanced Vanadium Redox Flow Batteries

Qi Deng, Wei-Bin Zhou, Hong-Rui Wang, Na Fu, Xiong-Wei Wu and Yu-Ping Wu*

Supporting Information

Aspergillus Niger Derived Wrinkle-like Carbon as Superior Electrode for Advanced Vanadium Redox Flow Batteries

Qi Deng, Wei-Bin Zhou, Hong-Rui Wang, Na Fu, Xiong-Wei Wu, Yu-Ping Wu*

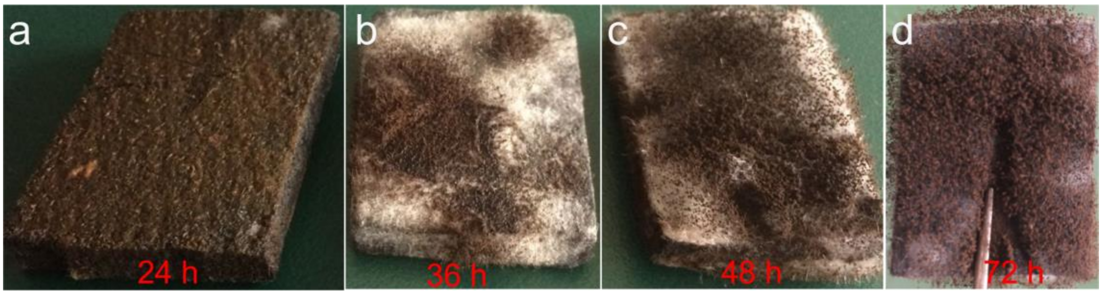

Figure S1. Optical images of the WLC electrode precursor at different culturing stages of: a) 24 h; b) 36 h; c) 48 h; d) 72 h.

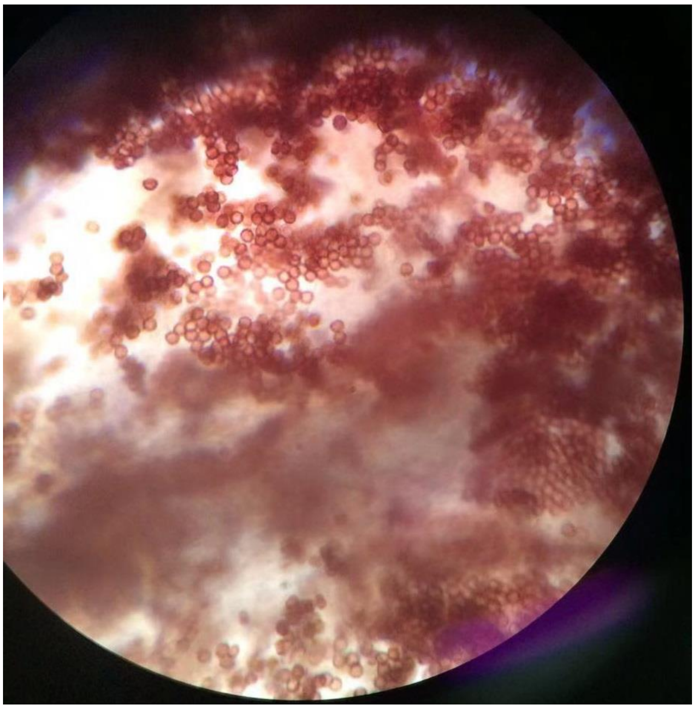

Figure S2. Optical image of *Aspergillus Niger* spores.

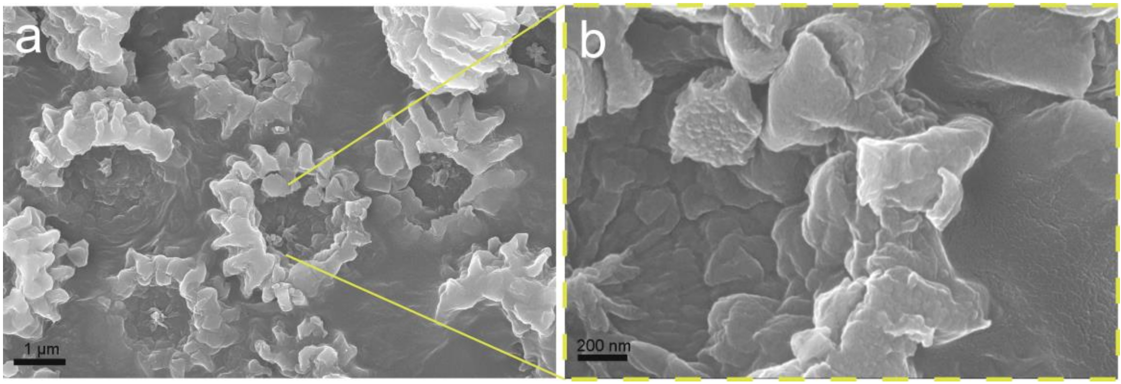

Figure S3. SEM images of *Aspergillus Niger* spores with different scale bar: a) 1 μm；b) 200 nm.

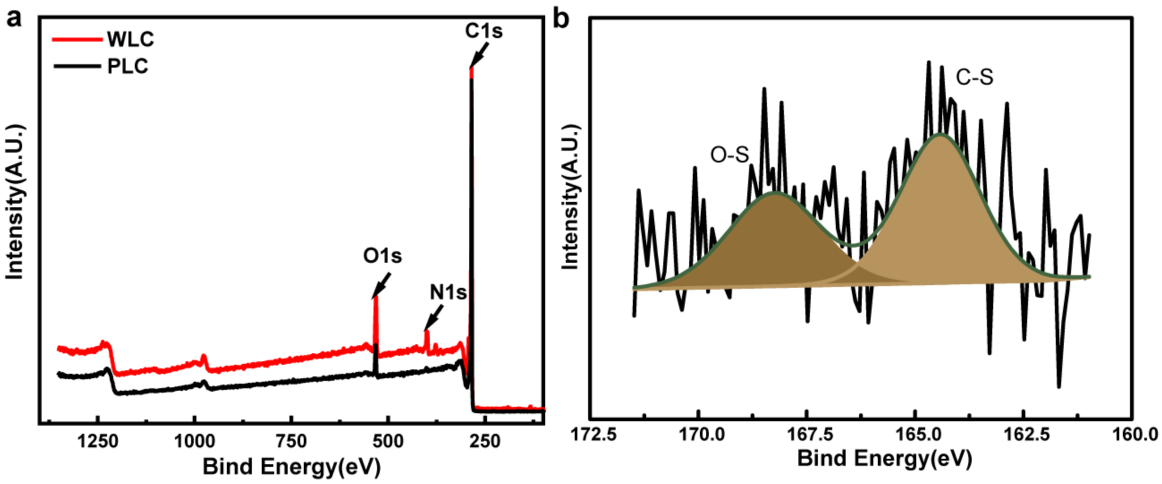

Figure S4. XPS spectra of: a) wide spectra of WLC and PLC materials. b) S 2p peaks and the high-resolution fitting of the WLC materials.

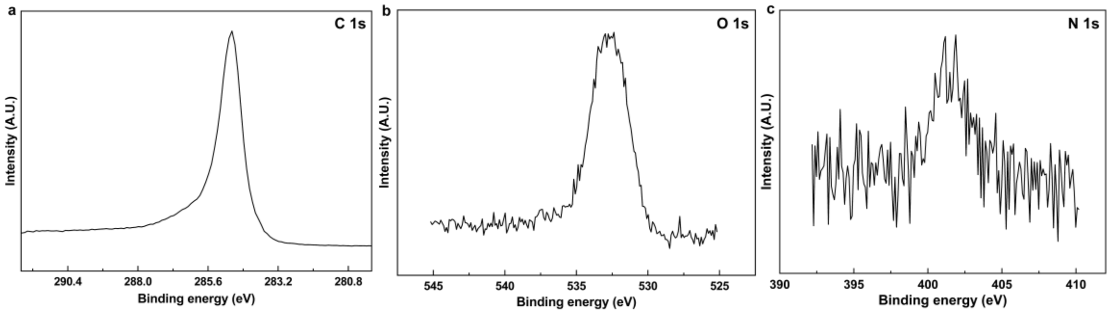

Figure S5. a) C 1s, b) O 1s, and c) N 1s peaks from the XPS of the PLC electrode.

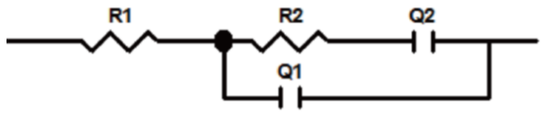

Figure S6. Equivalent circuit for the EIS spectra.

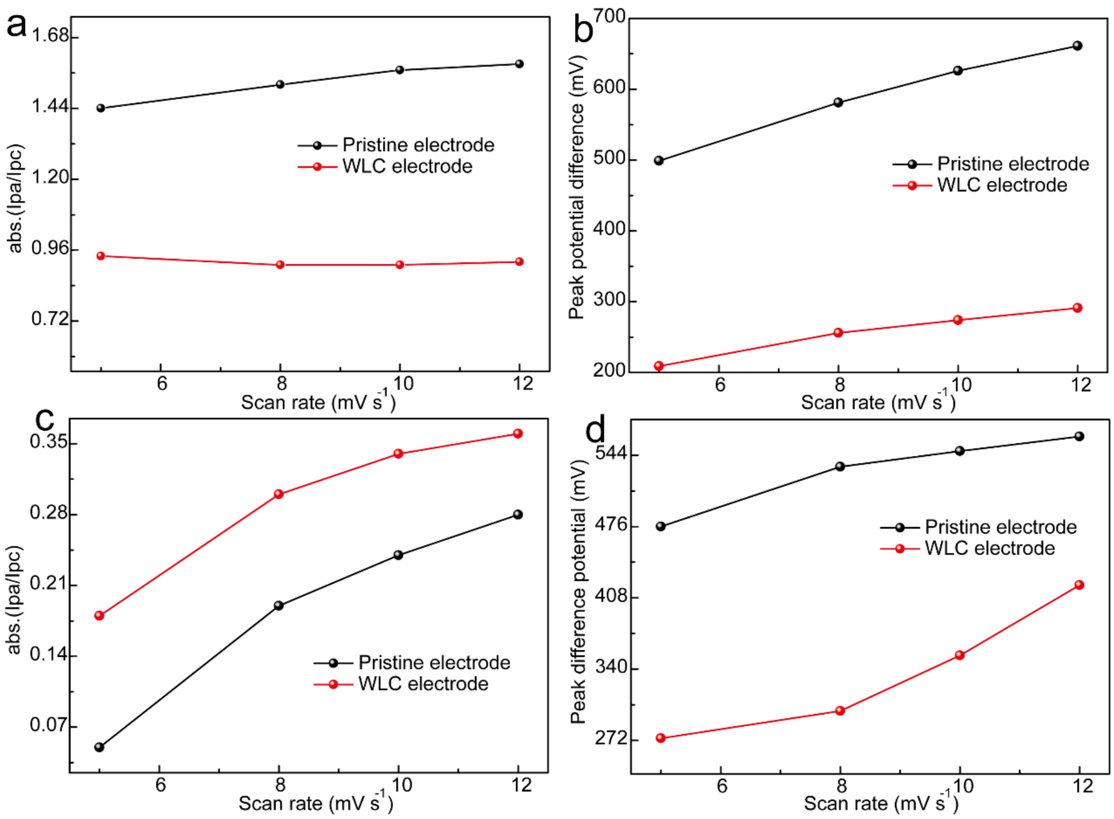

Figure S7. Plot of the (a, c) peak current ratios and (b, d) peak potential differences for the WLC and PLC electrode versus each scan rates (5, 8, 10, 12 mV s^{-1}).

Table S1. Electrochemical performances of the PLC and WLC electrodes derived from cyclic voltammetry tests for the V(II)/V(III) and V(IV)/V(V) redox couples.

		mA cm^{-2}		V			mV
	electrode	I_{pa}	I_{pc}	V_{pa}	V_{pc}	*Abs.* (I_{pa}/ I_{pc})	$\triangle E$
Positive half-cell	PLC	429.2	-173.1	1.265	0.725	2.48	540
	WLC	130.5	-85.5	1.080	0.797	1.53	283
Negative half-cell	PLC	82.3	-571.2	-0.288	-0.785	0.14	497
	WLC	67.2	-166.3	-0.317	-0.567	0.40	250

Abs.=Absolute value

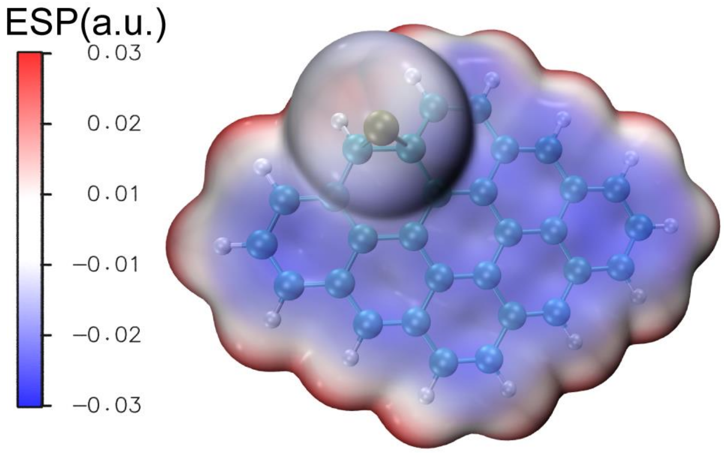

Figure S8. The ESP distribution on the Van der Waals surface of WLC with phosphorus atoms.

Table S2. Energy summaries of different systems: V^{2+}, PLC absorbed with V^{2+}, WLC absorbed with V^{2+}.

V^{2+}	**E_1 (a.u.)**	**E_2 (a.u.)**	**E_{total} (a.u.)**	**E_b (a.u.)**
**PLC**	-943.0179	-1151.9916	-2095.3716	0.3621
WLC	-943.0179	-1227.1719	-2170.6120	0.4221

Table S3. Energy summaries of different systems: V^{3+}, PLC absorbed with V^{3+}, WLC absorbed with V^{3+}.

V^{3+}	**E_1 (a.u.)**	**E_2 (a.u.)**	**E_{total} (a.u.)**	**E_b (a.u.)**
**PLC**	-941.9027	-1151.9916	-2094.8625	0.9682
WLC	-941.9027	-1227.1719	-2170.2068	1.1321

Table S4. Energy summaries of different systems: VO^{2+}, PLC absorbed with VO^{2+}, WLC absorbed with VO^{2+}.

VO^{2+}	**E_1 (a.u.)**	**E_2 (a.u.)**	**E_{total} (a.u.)**	**E_b (a.u.)**
**PLC**	-1018.3200	-1151.9916	-2170.6934	0.3818
WLC	-1018.3200	-1227.1719	-2245.8916	0.3996

Table S5 Energy summaries of different systems: VO_2^+, PLC absorbed with VO_2^+, WLC absorbed with VO_2^+.

VO_2^+	**E_1 (a.u.)**	**E_2 (a.u.)**	**E_{total} (a.u.)**	**E_b (a.u.)**
**PLC**	-1093.9991	-1151.9916	-2246.3181	0.3273
WLC	-1093.9991	-1227.1719	-2321.5617	0.3907

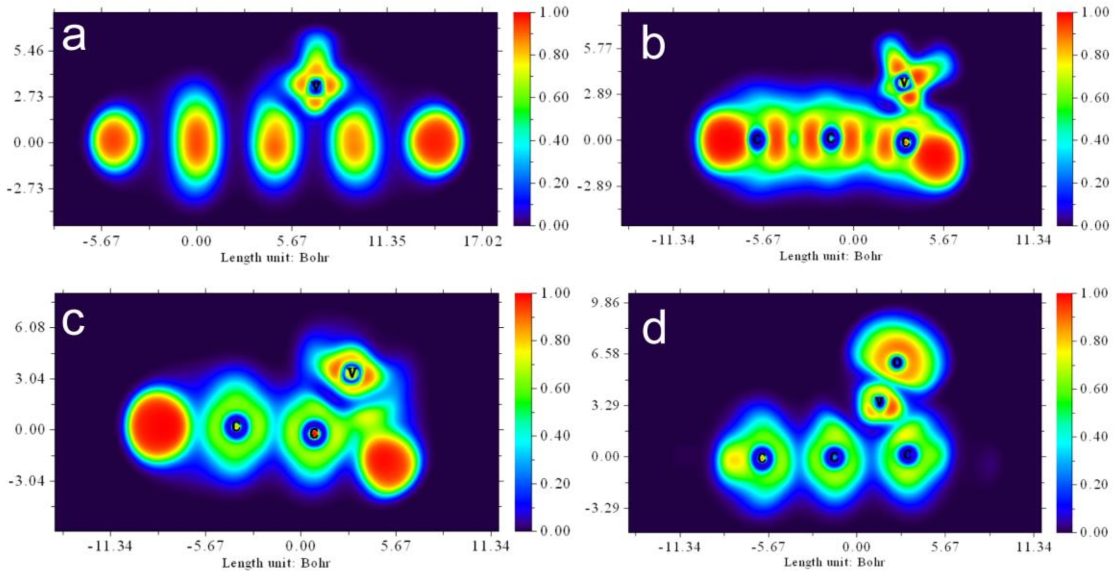

Figure S9. Electron localization function (ELF) plane of: a) PLC and c) WLC with V^{2+}; b) PLC and d) WLC with V^{3+}.
